# Case report: Acute *Talaromyces marneffei* mediastinitis in an HIV-negative patient

**DOI:** 10.3389/fmicb.2022.1045660

**Published:** 2022-11-11

**Authors:** Liangyu Chen, Meichun Zhang, Weihong Guo, Wenshuang Ding, Jinwen Tan, Hong Du, Ziwen Zhao, Weinong Zhong

**Affiliations:** ^1^Department of Respiratory and Critical Care Medicine, Zhuhai Hospital of Integrated Traditional Chinese and Western Medicine, Zhuhai, China; ^2^Faculty of Chinese Medicine, Macau University of Science and Technology, Macau, China; ^3^Department of Respiratory and Critical Care Medicine, Guangzhou First People's Hospital, Guangzhou, China; ^4^South China University of Technology, Guangzhou, China; ^5^Department of Pathology, Guangzhou First People's Hospital, Guangzhou, China

**Keywords:** mediastinal mass, nebulized amphotericin B, *Talaromyces marneffei*, mediastinitis, EBUS-TBNA, HIV-negative, immunocompetent

## Abstract

*Talaromyces marneffei* (*T. marneffei*) is one of the most important opportunistic human pathogens endemic in Southeast Asia. Talaromycosis, which was once regarded as an opportunistic infectious disease in patients with acquired immunodeficiency syndrome, is being increasingly reported in HIV-negative populations. Since *T. marneffei* infection can be localized or disseminated, patients may present with a variety of symptoms. However, mediastinal infection attributed to *T. marneffei* is extremely rare. We report the case of a 32-year-old female who manifested a large mediastinal mass and was eventually diagnosed as acute *T. marneffei* mediastinitis. The patient was HIV-negative and had no direct contact with intermediate hosts. We successfully managed to treat the patient with inhaled amphotericin B deoxycholate and observed lesion absorption in subsequent CT examinations. To our knowledge, this is the first published case of *T. marneffei* mediastinitis and first use of inhaled antifungal monotherapy on patients with *T. marneffei* infection.

## Introduction

*T. marneffei* was previously named *Penicillium marneffei* and the disease has variably been described as penicillosis or penicillosis marneffei. Among all discovered *Talaromyces* species, *T. marneffei* is the only thermally dimorphic species known to be pathogenic to humans. At 25°C, the colonies of *T. marneffei* are greenish-yellow, granular, and circular in shape, surrounded by characteristic red diffusible pigment. Little or no red pigment occur in the yeast phase at 37°C (Cooper, 1997). Microscopically, the mold form is like other *Talaromyces* species, with hyaline, septate and branched hyphae. Conidiophores give rise to three to five phialides, where chains of conidia are formed (Vanittanakom et al., 2006). The bamboo rat is considered as main natural reservoir host of *T. marneffei* (Deng et al., 1986). *T. marneffei* infections in humans are overwhelming in immunocompromised individuals, however a number of cases in apparent immunocompetent individuals have been described (Duong, 1996; Qiu et al., 2015; Pruksaphon et al., 2022). Increasingly these HIV-negative patients are found to have a variety of other immunocompromising conditions (You et al., 2021; Liu et al., 2022). Clinically, talaromycosis is usually referred to as an easily misdiagnosed, intractable and high-mortality disease, for its uncertain pathogenesis, non-specific symptoms, various imaging manifestations and complicated treatment with serious side effects. This case report aims to inform physicians about the clinical presentation, imaging study, pathological characteristics and treatment of *T. marneffei* mediastinitis.

## Case presentation

A 32-year-old woman presented with acute-onset shortness of breath and chest pain for 4 days. She also complained of cough with some small amounts of phlegm and occasional low-grade fever, but no night sweat, rash, hemoptysis, loss of weight, joint swelling, or any other discomfort. Her medical history included hypotension, hypoglycemia and bile reflux gastritis. She was also a hepatitis B virus carrier and once arranged for an interventional operation for spontaneous intracerebral hemorrhage at the age of 20. Luckily, no sequela was found after that operation. Her home medications included itopride, sucralfate and famotidine. She was a lifelong non-smoker and had no history of recent travel, wild animal contacts or sick contacts. She lived in Guangdong province in China. She had no known allergies.

After admission to hospital, physical examination revealed the following: blood pressure was 92 mmHg systolic and 65 mmHg diastolic, respiratory rate was 21 breaths/min, heart rate was 122 beats/min, body temperature was 99.7°F, arterial oxygen saturation on room air was 95%. She had coarse breath sounds on auscultation without any wheezing, rhonchi or crackles. The remainder of the physical examination produced normal results.

## Diagnostic studies

**Figure 1:**
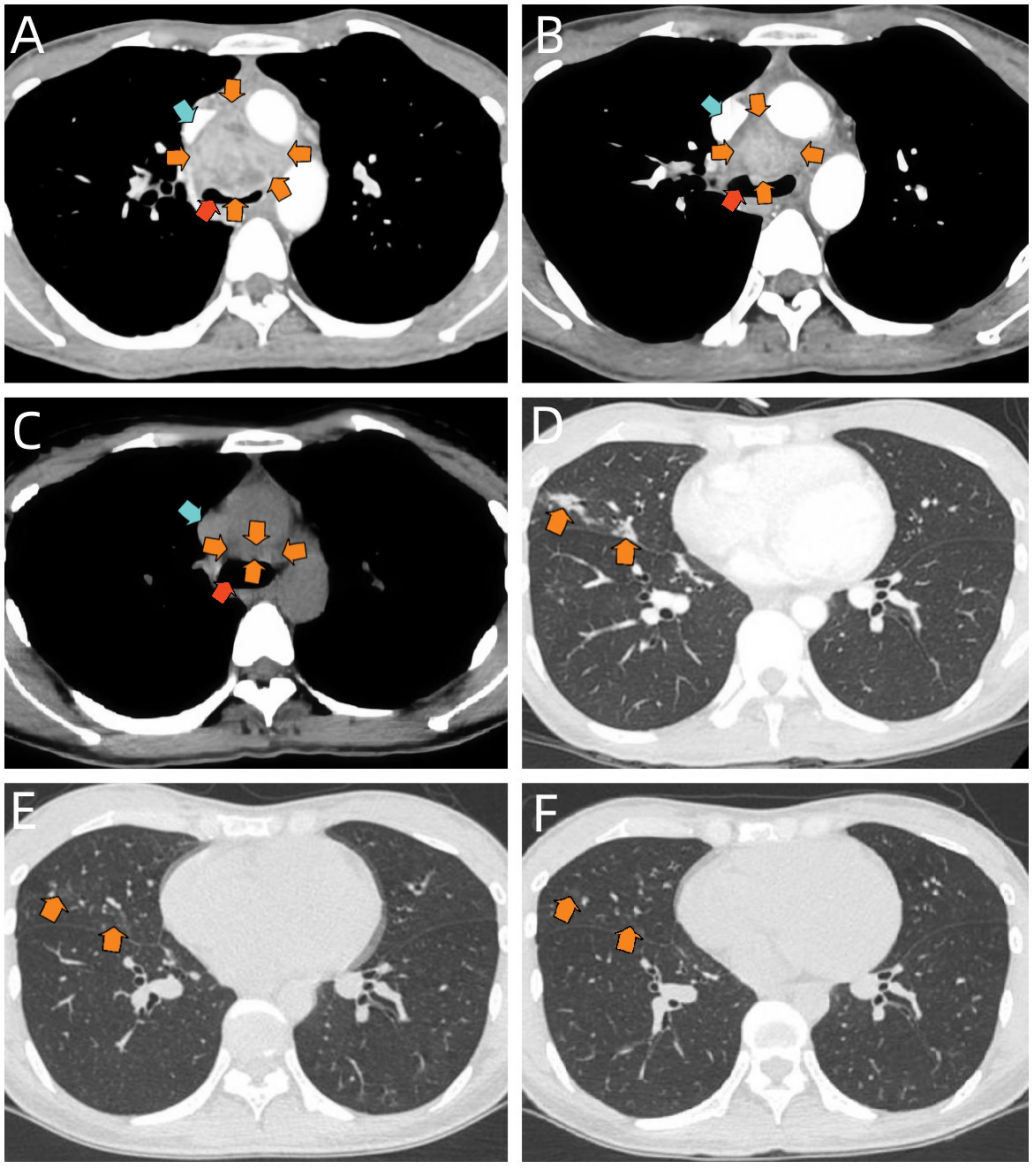
**(A)** Selected axial image of the soft-tissue window on contrast-enhanced chest CT before treatment, showing a mass in middle mediastinum (orange arrows) with compression of lower trachea (red arrow) and superior vena cava (blue arrow), it is heterogeneous in density. **(B)** Selected axial image of the soft-tissue window on contrast-enhanced chest CT after 2-week therapy of nebulized AmBd, suggesting the middle mediastinal mass shrinks (orange arrows) and the compression of lower trachea (red arrow) and superior vena cava (blue arrow) is partially relieved. **(C)** Selected axial image of the soft-tissue window on chest CT scan at 3-month follow-up, demonstrating the significant mass shrinkage in the middle mediastinum (orange arrows). The compression of lower trachea (red arrow) and superior vena cava (blue arrow) is almost completely relieved. **(D)** Selected axial image of the pulmonary window on chest CT before treatment, showing patchy infiltrates in the right middle lobe (orange arrows). **(E)** Selected axial image of the pulmonary window on chest CT after 2-week therapy of nebulized AmBd, suggesting the infiltrates in the right middle lobe are partially absorbed (orange arrows). **(F)** Selected axial image of the pulmonary window on chest CT at 3-month follow-up, demonstrating further absorption in the right middle lobar lesion (orange arrows).

Laboratory results suggested white blood cell count of 14,360 cells/mm (3) [normal value, 3,500–9,500 cells/mm(3)], neutrophil count of 10,820 cells/mm(3) [normal value, 1,800–6,300 cells/mm(3)], lymphocyte count of 1,960 cells/mm(3) [normal value, 1,100–3,200 cells/mm(3)], monocyte count of 850 cells/mm(3) [normal value, 100–600 cells/mm(3)] and procalcitonin level of 0.594 ng/ml (normal value, < 0.1 ng/ml). Blood gas analysis, NT-proBNP, D-dimer, cardiac enzymes were normal. T-Spot test suggested positive while other blood tests showed negative for blood culture, cryptococcal antigen latex agglutination test, candida galactomannan antigen test, (1-3)-β-D glucan antigen test, tumor-associated antigens and HIV antibodies. In terms of immune-related examinations, blood level of immunoglobulin G turned out 20.8 g/L, slightly above the normal value (8.6–17.4 g/L), while levels of immunoglobulin A, immunoglobulin M, immunoglobulin E, complement 3 and complement 4 were within normal range. Blood levels of 6 types of cytokines, TNF-α, IFN-γ, IL-2, IL-4, IL-6, and IL-10 were also normal. Moreover, no abnormal results were obtained on lymphocyte subset counts test, anti-extractable nuclear antigen antibodies test, antinuclear antibody test and vasculitis-associated autoantibodies test. Sputum culture and an electrocardiogram showed no obvious abnormalities. Initial chest radiograph indicated bilateral pneumonia. The contrasted chest CT demonstrated a 4.0 × 3.6 cm non-uniformly enhanced middle mediastinal mass with compression of lower trachea and superior vena cava (Figure 1A). Enlarged intramediastinal lymph nodes were appreciated, as well as right middle lobar infiltration (Figure 1D).

To obtain an accurate diagnosis, bronchoscopy, bronchoalveolar lavage (BAL), conventional transbronchial lung biopsy (TBLB) and endobronchial ultrasound-guided transbronchial needle aspiration (EBUS-TBNA) were performed on the 4th day of hospitalization.

**Figure 2:**
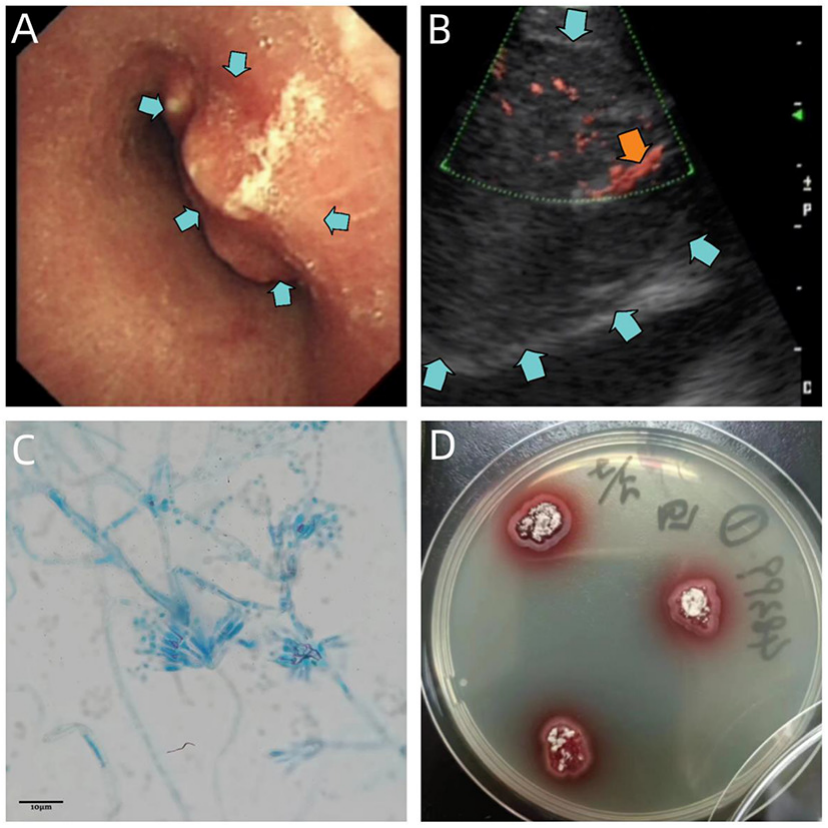
**(A)** Bronchoscopic view of the lower trachea, revealing severe compression stenosis caused by an extratracheal neoplasm (blue arrows). **(B)** Ultrasonogram of the extratracheal neoplasm reveals an enlarged para-aortic hypoechoic zone (blue arrows), with blood flow signals inside (orange arrow). **(C)** Micrograph of imprint smear obtained from cultured samples of needle aspirates at 25°C, suggesting conidiophores of *T. marneffei* bearing phialides and chains of conidia on the background of lactophenol cotton blue staining. Scale = 10 μm. **(D)** 5-day culture of needle aspirates of mediastinal mass at 25°C, demonstrating red-pigmented fungus colonies on Sabouraud dextrose agar plate.

**Figure 3:**
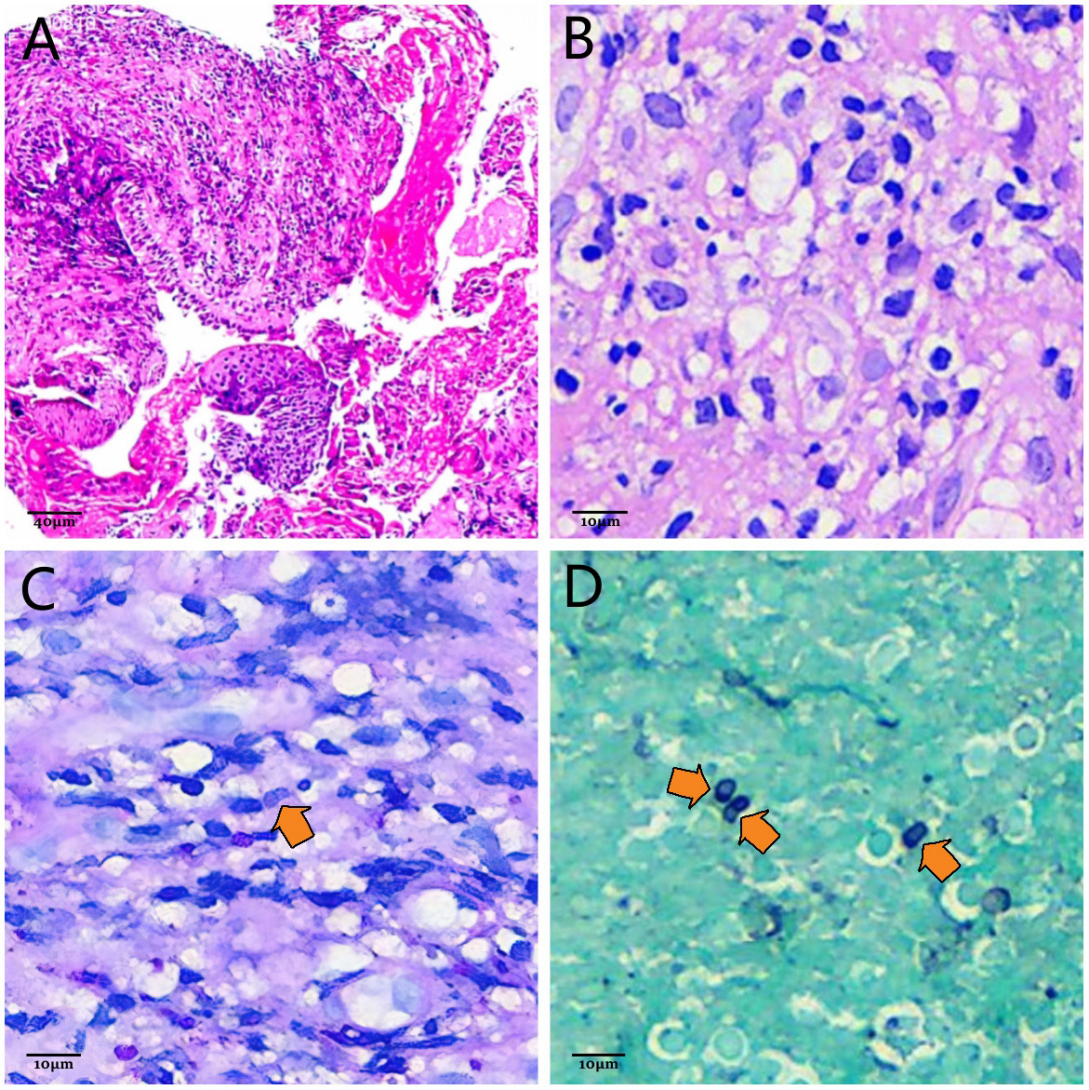
**(A)** Photomicrograph of TBLB. Infiltrations of massive neutrophils and lymphocytes are demonstrated by hematoxylin-eosin staining, which indicates acute inflammation (HEx200). Scale = 40 μm. **(B)** Photomicrograph of needle-aspirated material obtained by EBUS-TBNA with hematoxylin and eosin staining. Yeast cells of *T. marneffei* with transverse septa are difficult to observe on the background of acute inflammatory infiltration (HEx800). Scale = 10 μm. **(C)** Photomicrograph of needle-aspirated material obtained by EBUS-TBNA with PAS staining. The characteristic transverse septum within the sausage-shaped yeast cell (orange arrow) can be identified (PASx800). Scale = 10 μm. **(D)** Photomicrograph of needle-aspirated material obtained by EBUS-TBNA with GMS staining. Ovoid, elliptic and sausage-shaped yeast cells with characteristic transverse septa (orange arrows) are more distinguishable compared with PAS staining (GMSx800). Scale = 10 μm.

Bronchoscopy revealed severe trachea compression stenosis caused by an extratracheal neoplasm anterior to the lower trachea (Figure 2A), along with bronchial inflammation and airway secretion. Mixed bacteria were found yet indistinguishable by BALF smear and BALF culture. mNGS of BALF detected *T. marneffei* with 15 sequence reads identified out of 84 total reads. TBLB showed acute inflammation, negative for Acid-fast, PAS and GMS stains, and no tumor cells were seen (Figure 3A). Whereas, with the technology of EBUS-TBNA, we were able to observe the ultrasonogram of the extratracheal neoplasm (Figure 2B) and obtain the needle aspirates of the mass. On the 9th day of admission, culturing on Sabouraud dextrose agar medium at 25°C confirmed *T. marneffei* in the biopsied mass with colonies showing characteristic red-pigmented fungal colonies from the needle aspirates (Figure 2D). Under the microscope, the mold form of *T. marneffei* was also observed in imprint smear obtained from cultured samples of needle aspirates at 25°C on the background of lactophenol cotton blue staining (Figure 2C). On the same day, histopathologic picture of needle aspirates demonstrated acute exudative inflammation and focal necrosis, where yeast cells with characteristic transverse septa were identified through PAS and GMS staining (Figures 3C,D). Based on the evidence above, we finally proved the diagnosis was acute *Talaromyces marneffei* mediastinitis.

We initially treated the patient with intravenous moxifloxacin (dose: 400 mg/d), but the broad-spectrum antibiotic seemed to make little effect on her. Her shortness of breath and chest pain continued, while the cough and fever were even exacerbated. We changed the treatment into intravenous amphotericin B deoxycholate (AmBd) (initial dose: 0.1 mg/kg/d) immediately on the day when the diagnosis was confirmed. However, the patient experienced hypotension (BP: 70/43 mmHg) and refused to continue the therapy after the first infusion. Therefore, on the 10th day of hospitalization, she was treated with inhaled AmBd (dose: 0.7 mg/kg/d). As expected, the patient's symptoms were gradually alleviated during the therapy. She was eventually discharged with imaging improvement (Figures 1B,E) after 2-week treatment of nebulized AmBd. At 3-month follow-up, the patient was still stable with subsequent therapy of oral itraconazole (dose: 400 mg/d) and CT scan uncovered significant improvement (Figures 1C,F).

## Discussion

Although the mechanisms in the pathogenesis of *T. marneffei* are not fully understood, inhalation of conidia seems to be the main route of transmission (Pruksaphon et al., 2022). In China, most cases are reported in the southern part of the country, particularly Guangdong and Guangxi province, which indicates that talaromycosis is regionally related (Narayanasamy et al., 2021; Pruksaphon et al., 2022). Plenty of reports suggest talaromycosis in immunocompetent patients (Duong, 1996; Ye et al., 2015; Wang et al., 2017) but do not provide categorical evidence for immunocompetency. Recent studies have shown that some infected individuals thought to be non-immunosuppressed carry neutralizing anti-IFN-γ autoantibodies and associated HLA alleles (Guo et al., 2020). Additionally some HIV-negative patients have been shown to carry immune-related genetic mutations such as CD40L, STAT1, STAT3 and CARD9 (You et al., 2021; Liu et al., 2022). For this reason, in spite of the fact that there was no evidence suggesting this patient suffered from immune diseases, her immunocompetency remains undetermined. Genetic testing and the detection of anti–IFN-γ autoantibodies may have revealed a potential immune impairment, but unfortunately none of these had been arranged for this patient.

Since *T. marneffei* infection may have been localized or disseminated, clinical manifestations are varied according to different infection sites. Patients with *T. marneffei* chest infection are likely to suffer from fever, cough with little phlegm, dyspnea and chest pain (Duong, 1996; Wei et al., 2021). These symptoms are similar to our patient's and non-specific compared with other infectious mediastinitis and pneumonia. It indicates that the clinical presentation of *T. marneffei* mediastinitis plays a less important role in the diagnosis.

The images of *T. marneffei* chest infection also lack specificity. Resemble with pulmonary tuberculosis, *T. marneffei* chest infection can show a variety of lung abnormalities on chest CT imaging, including infiltration, nodules, cavity, ground-glass shadows, diffuse miliary shadows, pleural effusion and so on, usually along with enlargement in hilar and mediastinal lymph nodes (Shi et al., 2020; Wei et al., 2021). In this case report, it's also shown that *T. marneffei* chest infection can even manifest a large mediastinal mass on imaging when it involves the mediastinum. Based on the anatomical structure of mediastinum and radiologic manifestation of *T. marneffei* chest infection, we assume that the formation of the mediastinal mass may derive from the infection of mediastinal lymph nodes. For the similarity in radiography, physicians should differentiate *T. marneffei* mediastinitis from other mediastinal diseases, such as mediastinal lymphatic tuberculosis, lymphoma, thymoma and mediastinal teratoma.

We eventually diagnosed the patient by performing EBUS-TBNA to obtain mediastinal lesion samples. In contrast with conventional TBNA and surgery to access mediastinal lesions, the use of EBUS-TBNA in mediastinal diseases has become increasingly prevalent due to its simplicity of operator, minimal invasiveness and fewer adverse events. Researches show with consistency that EBUS-TBNA has high specificity and accuracy, while its sensitivity in diagnosis of mediastinal diseases is controversial. Despite that, it makes for the ideal first step in the diagnosis of pathology in mediastinal lesions (Divisi et al., 2018; Murthi et al., 2020).

Even though the patient's mNGS result proves its high-sensitivity and rapidness, it isn't considered as convincing evidence to make a definite diagnosis of *T. marneffei* infection as the result of its relatively low specificity (Zhang et al., 2022). The gold standard for diagnosis of *T. marneffei* is microbiological culture. It is typified by fungus colonies with massive production of red pigment at 25°C (Figure 2D). Microscopically, hyaline, septate and branched hyphae with conidiophores and conidia can be appreciated after incubation at 25°C (Figure 2C). Histologically, the transverse septum within the yeast cell is the most distinguishable feature of *T. marneffei* (Vanittanakom et al., 2006). However, with hematoxylin and eosin staining, yeast cells with the characteristic septated structure are usually difficult to identify on the background of infiltrations of neutrophils, lymphocytes and histiocytes (Figure 3B), which makes it hard to distinguish *T. marneffei* from *Histoplasma capsulatum* (Widaty et al., 2020). In contrast, once stained with PAS or GMS, especially GMS, yeast cells with transverse septa can have been more easily appreciated in histological observation (Figures 3C,D). Our patient was definitely diagnosed with *T. marneffei* mediastinitis according to the typical microbiological and pathological characteristics.

Though guidelines for *T. marneffei* infection in HIV-infected patients have been published (Kaplan et al., 2009; Nelson et al., 2011), standardized treatments for HIV-negative patients have not been established. In guidelines for HIV-positive patients, experts recommend intravenous infusion of AmBd as preferred treatment, but the adverse effects of intravenous AmBd make plenty of patients unable to continue the therapy. For example, the intravenous treatment may cause hypokalemia, hypotension, hyperpyrexia, arrhythmia, neurological symptoms and hepatic dysfunction. Besides, renal impairment has been seen among most patients (Saliba, 2006). Our patient failed to tolerate intravenous AmBd because she felt dizzy and her blood pressure dropped to 70/43 mmHg after the first infusion. As a result, we attempted to apply inhaled AmBd to the patient, for researches prove that nebulized AmBd can have been well-delivered in the bronchial and alveolar compartments with concentrations in BALF above most fungal MICs, and no or very weak systemic absorption is detected (Lowry et al., 2007; Brunet et al., 2022). These highlights of pharmacokinetics ensure nebulized AmBd to maintain its effect in the chest infection and limit its side reactions at the same time. Moreover, the use of nebulized AmBd is simple and convenient. Patients may prefer to accept aerosolized treatment of AmBd for its simplicity, as it reduces the frequency of liver and kidney function tests, serum potassium tests, routine blood tests and urine tests, which are necessarily required during the therapy of intravenous AmBd. Nevertheless, based on the fact that AmBd inhalation results in high alveolar concentrations and no or very low systemic absorption, the nebulized treatment may not be appropriate for disseminated talaromycosis nor extrathoracic *T. marneffei* infection. Physicians should also notice the side effects of inhaled AmBd, including cough, shortness of breath, difficulty breathing, chest tightness and bronchospasm (Lowry et al., 2007). In addition, liposomal amphotericin B may have been a better candidate for nebulization than AmBd, for it has higher drug concentration in the lungs and lower risk of toxicity (Allen et al., 1994; Ruijgrok et al., 2001).

## Conclusion

To sum up, when patients manifest a mediastinal mass in radiologic images, clinicians should consider mycotic mediastinal infection besides other common mediastinal diseases. EBUS-TBNA is the ideal first step in the diagnosis of mediastinal lesions. Aerosolized AmBd has the potential to become first-line treatment in HIV-negative patients with localized *T. marneffei* mediastinitis and pneumonia. However, as it's the first published case of nebulized AmBd monotherapy for *T. marneffei* chest infection, more studies and clinical trials are required.

## Data availability statement

The original contributions presented in the study are included in the article/supplementary material, further inquiries can be directed to the corresponding author/s.

## Ethics statement

Written informed consent was obtained from the individual(s) for the publication of any potentially identifiable images or data included in this article.

## Author contributions

LC contributed to the clinical design and concept. LC, MZ, and WG acquired the clinical data. JT and WZ performed clinical practices. WD and HD performed pathological analyses. LC, MZ, and ZZ interpreted the data and drafted and revised the manuscript. All authors discussed, read, approved the manuscript, and authorized its submission for publication.

## Conflict of interest

The authors declare that the research was conducted in the absence of any commercial or financial relationships that could be construed as a potential conflict of interest.

## Publisher's note

All claims expressed in this article are solely those of the authors and do not necessarily represent those of their affiliated organizations, or those of the publisher, the editors and the reviewers. Any product that may be evaluated in this article, or claim that may be made by its manufacturer, is not guaranteed or endorsed by the publisher.

## Abbreviations

BAL: bronchoalveolar lavage
BALF: bronchoalveolar lavage fluid
TBLB: conventional transbronchial lung biopsy
EBUS-TBNA: endobronchial ultrasound-guided transbronchial needle aspiration
TBNA: transbronchial needle aspiration
PAS: periodic acid-Schiff
GMS: grocott methenamine silver
mNGS: metagenomic next-generation sequencing
AmBd: amphotericin B deoxycholate
MIC: minimum inhibitory concentration.
